# RADIOGRAPHIC AND FUNCTIONAL EVALUATION OF HIGH POROUS METAL OSSEOINTEGRATION IN ACETABULAR REVISIONS WITH PELVIC DISSOCIATION

**DOI:** 10.1590/1413-785220253304e291300

**Published:** 2025-09-08

**Authors:** Reuder Pereira Prado, Lauro Barbosa, Gabriel Rodrigues Silva, Leandro Alves de Oliveira

**Affiliations:** 1 Clinica de Ortopedia e Traumatologia (COT), Goiania, GO, Brazil.; 2 Hospital das Clinicas da Universidade Federal de Goias, Goiania, GO, Brazil.

**Keywords:** Arthroplasty, Replacement, Hip, Radiography, Osseointegration, Acetabulum, Tantalum, Artroplastia de Quadril, Radiografia, Osseointegração, Acetábulo, Tantálio

## Abstract

**Introduction:**

Pelvic dissociation is a complex condition with a high rate of surgical complications and no established standard treatment. Surgery aims to preserve limb function by restoring bone structure and hip biomechanics. Conventional orthopaedic implants are made from materials such as stainless steel, cobalt-chrome or titanium. High-porosity implants have been introduced to improve the durability of hip arthroplasty.

**Objective:**

To evaluate osseointegration after revision total hip arthroplasty in cases of pelvic dissociation.

**Method:**

A descriptive observational study carried out at the Hospital das Clínicas of the Federal University of Goiás and at the Orthopedics and Traumatology Clinic (COT) in Goiânia. Data was collected from the medical records of patients who underwent total hip arthroplasty between 2012 and 2020, using titanium or tantalum implants, both high-porosity materials.

**Results:**

The study included 26 patients, 53.8% female and 46.2% male. The majority (34.6%) were aged between 60 and 69. Of the patients, 53.8% used titanium implants and 46.2% tantalum. After surgery, 38.5% had a Harris Hip Score between 70 and 80, with 61.5% of patients showing osseointegration in less than six months.

**Conclusion:**

Tantalum, due to its high porosity, was effective in treating patients with pelvic dissociation undergoing revision total hip arthroplasty. ***Level Of Evidence lV; Descriptive Observational Study***.

## RESUMO

**Introdução:**

A dissociação pélvica é uma condição complexa com alta taxa de complicações cirúrgicas e sem um tratamento padrão estabelecido. A cirurgia visa preservar a função do membro ao restaurar a estrutura óssea e a biomecânica do quadril. Implantes ortopédicos convencionais são feitos de materiais como aço inoxidável, cobalto-cromo ou titânio. Os implantes de alta porosidade foram introduzidos para melhorar a durabilidade da artroplastia do quadril.

**Objetivo:**

Avaliar a osteointegração após revisão de artroplastia total de quadril em casos de dissociação pélvica.

**Método:**

Estudo observacional descritivo realizado no Hospital das Clínicas da Universidade Federal de Goiás e na Clínica de Ortopedia e Traumatologia (COT) em Goiânia. Foram coletados dados de prontuários de pacientes que realizaram artroplastia total de quadril entre 2012 e 2020, utilizando implantes de titânio ou tântalo, ambos materiais de alta porosidade.

**Resultados:**

O estudo incluiu 26 pacientes, 53,8% do sexo feminino e 46,2% do masculino. A maioria (34,6%) tinha entre 60 e 69 anos. Dos pacientes, 53,8% utilizaram implantes de titânio e 46,2% de tântalo. Após a cirurgia, 38,5% apresentaram Harris Hip Score entre 70 e 80, com 61,5% dos pacientes mostrando osteointegração em menos de seis meses.

**Conclusão:**

O tântalo, devido à sua alta porosidade, foi eficaz no tratamento de pacientes com dissociação pélvica submetidos à revisão de artroplastia total de quadril. ***Nível de evidência IV; Estudo Observacional Descritivo***.

## INTRODUCTION

Pelvic dissociation is an uncommon condition that usually occurs in the total hip arthroplasty review population. Having increased incidence due to the number of primary hip total arthroplasties (HTA) and the need for revisions. The goal of the surgical treatment is to preserve the restoring function of the limb, bone structure and hip biomecany.^1-3^ Currently, HTA is the gold standard for this type of health condition, with excellent clinical results and long-term survival. However, the growth of this procedure corroborates the increase in failures.^1,4^

The acetabular review is indicated for patients who have progressive osteolysis, severe wear or bone loss that may compromise future reconstruction, including symptomatic aseptic release, failure to fix, infection, wear, osteolysis and instability, given the loss of the acetabular bone structure and the condition of soft tissues, the acetabular review represents a procedure that requires more attention.^4,5^

Bone defects involving less than 50% of the acetabulum are considered mild to moderate. Defects involving more than 50% are defined as massive. Biological fixation in cases of hip review with moderate pelvic bone defects requires sufficient contact between the bone and the acetabular dome, thus allowing primary stability and bone growth. A 50% contact between a porous dome and native bone is considered the bottom limit for reliable reconstruction using these devices. Less extensive surface contacts with native bone can be accepted if there is a good support with the edge or dome of the acetabula.^1^

The use of X-rays helps predict the severity and location of bone loss and guide treatment options, some classification systems are used to assist the surgeon in surgical planning, as in the case of the acetabular classification of *Paprosky* in total hip arthroplasty with failure, being based on the severity of bone loss and the ability to obtain cementless fixation for a certain pattern of bone loss.^4^

Orthopedic implants are generally made of stainless steel, cobalt-chrome (CoCr) or titanium alloys. Hip implants with high porosity coating were introduced about 30 years ago with the aim of improving the durability of hip arthroplasty. Initially used were sintered spheres of chrome-cobalt, diffusion-linked metal fiber mesh, sponge-structured titanium and titanium plasma spray. These materials allow bone growth inward or outward and remodel in the metal-bone interface, and longer rigid fixation. However, in acetabular review surgery, these materials demonstrate limitations when the native bone surface available for osteointegration is minimal.^4,6-9^

High porosity metal is a metal with special porous structures, which can offer high biocompatibility and low Young module to satisfy the need for orthopedic applications. Titanium and tantalum are the high-porosity metals most used in orthopedics due to their bio-mechanical properties and biocompatibility.^10^

High-porosity metals have been developed to improve the properties of the biomaterial of uncemented implants. The open cell structure of these materials is ideal for acetabular review surgery due to high volumetric porosity, low module elasticity and high friction characteristics. The new generation of high porosity metals has characteristics that allow bone consolidation and high osteointegration of metal implants.^4,5,11^

The main characteristics of an ideal high porosity metal require normal cellular activity without any local and systemic toxic effects on the host tissue, which offer mechanical properties similar to the host bone with sufficient mechanical strength, the seats must have macro (porous size > 100 mm) and microporosity (porous size < 20 mm) and the pores must be interconnected, have initial resistance to safe handling, be reproducible and processable in a three-dimensional structure and must tolerate sterilization according to the international standards required for clinical use, in addition to having reasonable manufacturing cost.^7,11^ The main disadvantage of metallic biomateria is the lack of biological recognition on the surface of the material, another limitation is possible release of ions and/or metallic particles by toxic corrosion or discharge by possible inflammatory cascades and allergic reactions.^7,11^

Metals such as tantalum and titanium have been commonly used.as bone substitutes or implants in orthopedic surgery because they present excellent corrosion. The high porosity tantalum has a biological performance equivalent to traditional high porosity titanium implants in repairing small bone defects.^12^ The tantalum has a elasticity module (GPa) between 2.5 and 3.9, mean pore size at 550 μm, 75% porosity and 0.88 friction coefficient. Titan has a elasticity module (GPa) between 106 - 115, average pore size at 616 μm, 60% porosity and 0.65 friction coefficient.^4,13^

Titanium alloys were first used in orthopaedics around 80 years ago and continue to be used because of their unique properties, including high specific strength, low weight, excellent corrosion resistance and biocompatibility.^14^ However, for bone replacement components, the strength of pure titanium is not enough and alloys of this material are used because of their superior mechanical properties, but it is a low porosity coating, ranging from 30-50%, which limits the maximum interfacial force that can be formed by bone growth.^11,15^ Titanium in the form of metal foam is indicated in the manufacture of high porosity metal implants in primary and revision total hip, knee and shoulder arthroplasty. The use of high porosity titanium acetabular components in cases of total hip arthroplastia and review is associated with satisfactory clinical outcomes in the short and medium term.^16^

Tantalum has been introduced in an effort to increase the osseointegration potential of uncemented components.^15^ High porosity tantaum is a tantalum structure of repeated dodecahedron open cells with an appearance similar to spongy bone. It has excellent biocompatibility and is safe for *in vivo* use. The bioactivity and biocompatibility of high porosity tantalum derive from their ability to form a superficial layer of self-passive oxide.^6,13^

Based on the above, the objective of the present study is to evaluate the post-revision osteointegration of total hip artroplasty in cases of pelvic dissociation, in addition to verifying the performance of the acetabular component with high porosity metal in postoperative functional evaluations.

## MATERIAL AND METHOD

Observational and descriptive research was conducted to evaluate osteointegration after review of total quadrillary arthroplastic in cases of pelvic dissociation in the Department of Orthopaedia and Traumatology of the Hospital of the clinics of the Universidade Federal de Goiás and the Clínica de Ortopedia e Traumatologia – COT, Goiânia.

Participants of both sexes, older than 18 years of age who had performed the Quadril Total Arthroplastia review between January 2012 and December 2020 and who had placed implants of high porosity material, titanium or tantalum, were included in the Department of Orthopaedia and Traumatology of the Hospital das Clínicas of the Universidade Federal de Goiás and the Clínica de Ortopedia e Traumatologia – COT, Goiânia. The data were collected the information concerning the place of surgery, Age, Gender, Color, Occupation, Comorbidities, Date of surgery, Material used, Surgery performed, Harris Hip Score before surgery, to evaluate the pain, Harris Hip score after surgery and time of osteointegration of the journal of the selected research participants.

For the analysis of the tabulated data, the IBM SPSS statistical analysis platform, version 21 was used. The confidence interval for this study was 95%, and the significance level of 0.05 was accepted. In these comparisons, p-values above 0.050 are considered not significant for this study as they are outside the desired confidence interval. The t-student test and the Qui-square test were used to verify whether the frequency of observed data in a question deviates significantly or not from the frequency with which it is expected and to compare the distribution of the data for different variables, in order to verify whether the observed proportions show significant differences or whether the samples differ significantly in the proportions of these variables, being correlated the type of material used (titanium or tantalum) with the best benefit in relation to post-Revision of Total Quadril Arthroplasty in Cases of Pelvic Dissociation.

This study was registered under CAEE number: 46384621. 6.0000.5078, and approved by the Research Ethics Committee of the Federal University of Goiás Clinic Hospital.

## RESULTS

41 patients were selected, 36.58% of which were excluded because there was no pelvic dissociation, with 26 (63.41%) research participants included in our study, the highest frequencies of participants included had the surgeries between the years of 2018 (42.3%), 2014 (15.4%), 2015 (11.5%) and 2017 (11.5%). In terms of sex, 53.8% were female and 46.2% were male. Furthermore, 34.6% were aged 60 to 69, 23.1% were aged 80 or above and 19.2% were aged 70 to 79. In relation to the profession, 57.7% were retired, 23.1% were paid external workers and 15.4% were public officials. Of the total, 30.8% of patients had some comorbidity.

In relation to the site of the surgery in our institution, 80.8% was performed at COT. Of the total, 53.8% of patients used Titanium and 46.2% Tantalum. Furthermore, 100% of patients performed hip arthroplasty review surgery, 100% of patients had Harris Hip Score before surgery less than 70 points, after surgery, 38.5% had Harris Hip Score between 70 to 80 points and 34.6% between 80 to 90. The most frequent osteointegration time was less than six months in 61.5% of the patients, in 11.5% of the patients included there was no osteointegration.

In relation to the correlation of the materials used in surgeries with the other variables, it was possible to observe that the patients who had the use of tantalum, 58.33% were female, 58.33% were retired, 25% had comorbidities, 33.33% had ages between 60 and 69 years and 25% had ages equal to or above 80 years. Of the total, 33.33% had the surgery in 2014 and 25% in 2015 and 91.67% had the surgery in COT. It was observed that 100% had Harris Hip Score before surgery less than 70 points, after surgery, 58.33% of these patients had Harris Hip Score between 80 to 90 points and 41.67% between 70 to 80 points (Table 1). Of the patients who used Titanium, 50% were female and 50 male, 57.14% were retired, 35.71% had comorbidities, 35.71% were aged 60 to 69 years, 21.43% were aged 70 to 79 years and 21.43% were aged 80 years or older. 64.29% had the surgery in 2018 and 71.43% had the surgery in COT. Furthermore, 100% had Harris Hip Score less than 70 points before surgery, after surgery, 35.71% of these patients had Harris Hip Score between 70 and 80 points, 50% of patients who had this type of material used in surgery continued with Harris Hip Score less than 70 points after surgery. (Table 1)

**Table 1:** Correlation of the material used with the other variables.

	Material used				Total		p-value
	Tantalum		Titanium				
Sex							
Female	7	(58.33%)	7	(50.00%)	14	(53.85%)	0.671
Male	5	(41.67%)	7	(50.00%)	12	(46.15%)	
Total	12	(46.15%)	14	(53.85%)	26	(100.00%)	
Declared profession							
retired	7	(58.33%)	8	(57.14%)	15	(57.69%)	0.458
From home	0	(0.00%)	1	(7.14%)	1	(3.85%)	
Public official	3	(25.00%)	1	(7.14%)	4	(15.38%)	
Paid external work	2	(16.67%)	4	(28.57%)	6	(23.08%)	
Total	12	(46.15%)	14	(53.85%)	26	(100.00%)	
Comorbidities							
Yes	3	(25.00%)	5	(35.71%)	8	(30.77%)	0.555
No	9	(75.00%)	9	(64.29%)	18	(69.23%)	
Total	12	(46.15%)	14	(53.85%)	26	(100.00%)	
Age of the patient at the time of surgery							
40 to 49 years	1	(8.33%)	1	(7.14%)	2	(7.69%)	0.997
50 to 59 years	2	(16.67%)	2	(14.29%)	4	(15.38%)	
60 to 69 years	4	(33.33%)	5	(35.71%)	9	(34.62%)	
70 to 79 years	2	(16.67%)	3	(21.43%)	5	(19.23%)	
>= 80 years	3	(25.00%)	3	(21.43%)	6	(23.08%)	
Total	12	(46.15%)	14	(53.85%)	26	(100.00%)	
Date of surgery							
2012	1	(8.33%)	0	(0.00%)	1	(3.85%)	0.019*
2014	4	(33.33%)	0	(0.00%)	4	(15.38%)	
2015	3	(25.00%)	0	(0.00%)	3	(11.54%)	
2016	1	(8.33%)	0	(0.00%)	1	(3.85%)	
2017	1	(8.33%)	2	(14.29%)	3	(11.54%)	
2018	2	(16.67%)	9	(64.29%)	11	(42.31%)	
2019	0	(0.00%)	1	(7.14%)	1	(3.85%)	
2020	0	(0.00%)	2	(14.29%)	2	(7.69%)	
Total	12	(46.15%)	14	(53.85%)	26	(100.00%)	
Local surgery							
COT	11	(91.67%)	10	(71.43%)	21	(80.77%)	0.192
HGG	1	(8.33%)	4	(28.57%)	5	(19.23%)	
Total	12	(46.15%)	14	(53.85%)	26	(100.00%)	
Harris Hip Score before surgery							
< 70 points	12	(100.00%)	14	(100.00%)	26	(100.00%)	-
70 to 80 points	0	(0.00%)	0	(0.00%)	0	(0.00%)	
80 to 90 points	0	(0.00%)	0	(0.00%)	0	(0.00%)	
90 to 100 points	0	(0.00%)	0	(0.00%)	0	(0.00%)	
Total	12	(46.15%)	14	(53.85%)	26	(100.00%)	
Harris Hip score after surgery							
< 70 points	0	(0.00%)	7	(50.00%)	7	(26.92%)	0.008*
70 to 80 points	5	(41.67%)	5	(35.71%)	10	(38.46%)	
80 to 90 points	7	(58.33%)	2	(14.29%)	9	(34.62%)	
90 to 100 points	0	(0.00%)	0	(0.00%)	0	(0.00%)	
Total	12	(46.15%)	14	(53.85%)	26	(100.00%)	
Osteointegration time							
< 6 months	9	(75.00%)	7	(50.00%)	16	(61.54%)	0.196
>= 6 months	3	(25.00%)	4	(28.57%)	7	(26.92%)	
There was no osteointegration	0	(0.00%)	3	(21.43%)	3	(11.54%)	
Total	12	(46.15%)	14	(53.85%)	26	(100.00%)	

* Statistically representative difference.

**Source:** Data collected by the authors.

## DISCUSSION

During the development of this study, we observed that post-revision osteointegration of total quadrillary arthroplastic in cases of pelvic dissociation was most effective after six months of the surgical process in most patients.

Regarding the materials used, we observed that tantalum in our institution, during the study period, is used with predominance in female patients and Harris Hip Score before surgery less than 70 points, after which, more than half of the patients had Harris Hip Score between 80 and 90 points. The use of titanium also had predominance in the female sex, Harris Hip Score before surgery less than 70 points, however, only slightly more than a third of these patients had the Harris Hip Score between 70 and 80 points. Patients who used titanium had a higher predominance of Harris Hip Score after surgery proceeding below 70 points.

Patients who used titanium also demonstrated low efficacy of osteointegration, with just over a fifth of them not having osteointegration. Although the use of tantalum was most effective in osteointegration, 3/4 of patients had osteointegration before six months, and 1/4 after this period.

**Figure 1:**
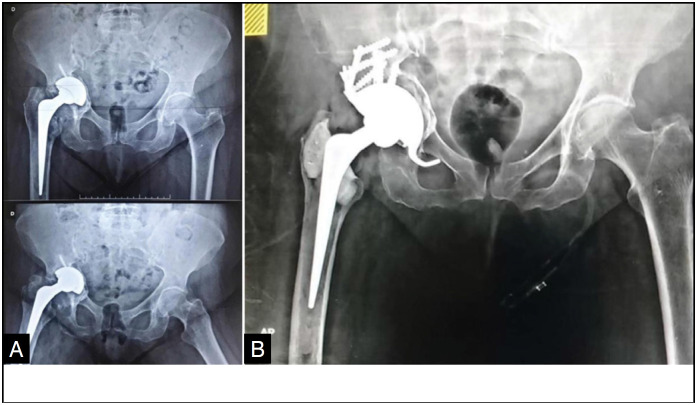
Case 01 (Image A: Preoperative X-ray and image B: X-ray three months after surgery).

Source: Prontuary of the patient (Service database).

**Figure 2:**
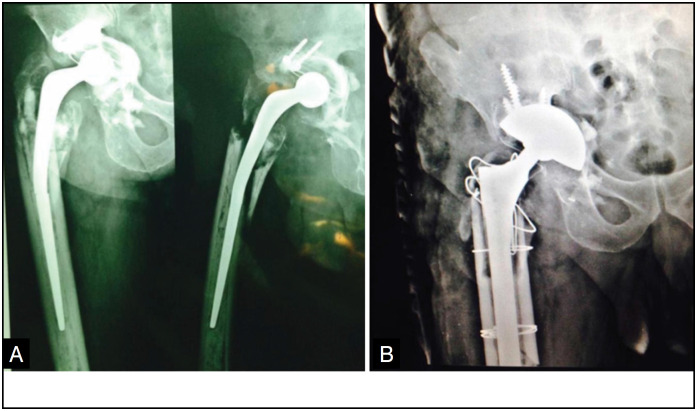
Case 02 (Image A: Preoperative X-ray and image B: X-ray six months after surgery).

Source: Prontuary of the patient (Service database).

In our study, the MBP patient, 80 years old, female, white, retired, with pelvic dissociation, performed Revision of hip artroplasty on the right side with titanium material in the COT institution, developed osteointegration in four months of postoperative, with Harris Hip Score preoperative of 38 points, and in the postoperative with 92 points (Figure 1). The MRN patient, 80 years old, male, white, retired, with pelvic dissociation, performed a review of hip artroplasty on the right side with tantalum material in the COT institution, developed bone integration in six months of postoperative, with Harris Hip Score preoperative of 30 points and in the postoperative with 82 points. (Figure 2)

This osteointegration period of less than six months in patients who used tantalum demonstrates that this material corroborates the patient's prior return to their activities. Various studies in the literature have demonstrated high survival rates after two years of treatment,^13,17-20^ as well as low infection rates^21^ in patients who used this material.

Despite the high rate of non-osteointegration in our study with use of titanium even after six months of follow-up, some studies in the literature have demonstrated that titanium use leads to satisfactory clinical and radiographic results for reconstruction of the acetabular defect in primary HTA in the long term.^22^ However, one study presented a case showing the risk of full and rapid wear of a ceramic femoral head through a polyethylene coating and titanium acetabular cup as well as signs of significantly elevated serum titanium ion levels.^16^

## CONCLUSION

After the development of this study we can observe that tantalum has had greater efficiency in osteointegration in a period of less than six months. The use of titanium showed a higher rate of non-osteointegration even over a period of more than twelve months. In relation to the Harris Hip score after surgery, patients using tantalum had an improvement in their consideration of score, from score less than 70 points before surgery to score between 80 and 90 after surgery. Patients who used titanium had greater retention at scores lower than 70 points after surgery. Thus, we can conclude that tantalum is a highly porous material that is effective in the treatment of patients after review of total hip artroplasty in cases of pelvic dissociation.
